# Substituent Effects of Phenyl Group on Silylene Bridge in Stereospecific Polymerization of Propylene with *C*_1_-Symmetric *Ansa*-Silylene(fluorenyl)(amido) Dimethyl Titanium Complexes

**DOI:** 10.3390/polym10101075

**Published:** 2018-09-28

**Authors:** Huajin Wang, Yanqing Li, Zhengguo Cai

**Affiliations:** 1 State Key Laboratory for Modification of Chemical Fibers and Polymer Materials, College of Material Science and Engineering, Donghua University, 2999 North Renmin Road, Shanghai 201620, China; liyanqingky@sina.com; 2 College of Materials and Textile Engineering, Jiaxing University, 118 Jiahang Road, Jiaxing 314001, China

**Keywords:** *C*_1_-symmetric, titanium complex, propylene polymerization, stereospecific

## Abstract

A *C*_1_-symmetric (methylphenyl)silylene-bridged (fluorenyl)(naphthylamido) titanium complex (**1**) and (diphenyl)silylene-bridged (fluorenyl)(naphthylamido) titanium complex (**2**) were synthesized and characterized by ^1^H NMR, element analysis, and X-ray crystal analysis. The coordination mode of the fluorenyl ligand to the titanium metal is an η^3^ manner in each complex. These complexes were applied for propylene polymerization using dried modified methyaluminoxane (dMMAO) as a cocatalyst under different propylene pressures in a semi batch-type method. The catalytic activity was strongly dependent on the structure of the complex and the propylene pressure, where complex **1** exhibited the highest activity (600 kg mol^−1^·h^−1^) under a propylene pressure of 8.0 atm to produce high molecular weight polypropylene. The polypropylenes obtained were syndiotactic-rich with an rr value of 0.50, indicating that the silylene bridge was not efficient for the isospecificity of a constrained geometry catalyst (CGC). The mechanical properties of the resulting polymers depended on their microstructure.

## 1. Introduction

**Scheme 1:**
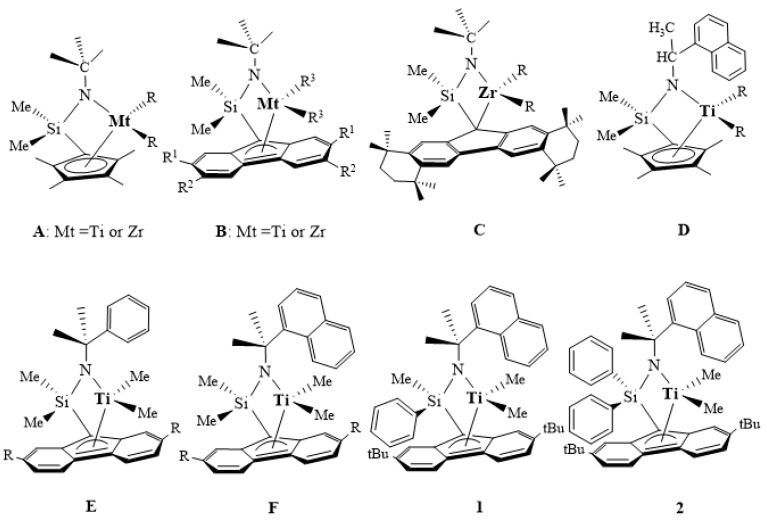
Chemical diagrams of mono-metallocene group 4 complexes.

Numerous homogeneous Ziegler–Natta catalysts based on metallocene were extensively developed as olefin polymerization catalysts to control the microstructure of polyolefins, such as molecular weight and molecular weight distribution, stereospecificity, copolymer composition, etc. [1,2,3,4,5,6,7,8,9,10]. Among them, constrained geometry catalysts (CGCs, **A**, Scheme 1) [11,12,13] containing an *ansa*-monocyclopentadienyl amido ligand have attracted significant attention due to their unique characteristics, including excellent copolymerization ability [14,15,16,17,18,19,20,21,22,23,24], stereospecificity [25,26,27,28,29], and living polymerization [30,31], which are all strongly dependent on a slight change of the ligand. The replacement of the cyclopentadienyl ligand by using a fluorenyl ligand (B, Scheme 1) improved both the activity and syndiospecificity [25,27], and the dimethyltitanium complexes were used to conduct living (co)polymerization of olefin [30,31]. The introduction of a butyl group on the fluorenyl ligand led to a very positive effect on improving the syndiospecificity [25,32]. Furthermore, sterically expanded zirconium complex (C, Scheme 1) exhibited the highest syndiospecificity (syndiotactic pentad (rrrr) > 0.99) [27].

The substituent effects on the amido ligand were also investigated. *C*_1_-symmetric complexes (D, Scheme 1) bearing sterically bulkier naphthyl amido ligand yielded isospecific-rich polypropylene (PP) with an isotactic triad (mm) of 0.61 [33,34]. In contrast to *C*_s_-symmetric CGC complexes, fluorenyl-ligated *C*_1_-symmetric complexes (E and F, Scheme 1) produced statistically atactic PP, indicating the different substituent effect of the fluorenyl ligand in the isospecific polymerization of propylene [35]. We supposed that two diastereotopically distinct sites, which are necessary for high isospecificity, were not provided in these *C*_1_-symmetric complexes by the fast rotation of the amido ligand. In this study, we thus synthesized (methylphenyl)silylene or (diphenyl)silylene-bridged *C*_1_-symmetric fluorenyl-naphthylamido dimethyltitanium complexes (**1** and **2**, Scheme 1) to investigate the substituent effects on the silylene bridge in the stereospecific polymerization of propylene.

## 2. Experimental Section

### 2.1. Materials

All operations were performed using standard Schlenk techniques. All solvents were distilled or purified by the PS-MD-5 (Innovative Technology (China) Ltd., HongKong, China) solvent purification system. Research-grade propylene was purified by a dehydration column of ZHD-20 and deoxidation column of ZHD-20A. Modified methylaluminoxane (MMAO) solution in toluene was donated by Tosoh-Finechem Co. (Shunan, Japan) Trialkylaluminium-free dried modified methylaluminoxane (dMMAO) was prepared from the toluene solution of MMAO by vacuum-drying followed by washing with hexane, as reported previously [32]. All the other reagents were purchased and used as received.

### 2.2. Synthesis of Titanium Complexes

#### 2.2.1. Synthesis of (Methylphenyl)silylene-Bridged Ligand L1

Step A. *n*-BuLi (2.5 M in hexane 2.8 mL, 7 mmol) was added dropwise at 0 °C into a solution of 1.3 g (7 mmol) of (dimethyl)(naphthyl)amine in 50 mL of diethylether, and the reaction mixture was stirred for 3 h at r.t.

Step B. The product obtained in step A was added at r.t. to a solution of 3.0 g (7 mmol) of (2,7-di-*t*BuFlu)(MePh)SiCl in 60 mL of diethylether, and the resultant yellow suspension was stirred overnight at r.t. The solvent was removed in vacuo, and the residue was extracted with hexane. The removal of the hexane gave (2,7-di-tBuFlu)(MePh)Si-naphthylamido as an orange solid in an 82.6% yield (3.4 g).

^1^H NMR (CDCl_3_, 7.26): 8.85 (d, 1H, Naph), 7.85 (d, 1H, Naph), 7.65 (d, 1H, Naph), 7.49 (d, 1H, Naph), 7.22 (t, 3H, Naph; 3H, Flu), 7.14 (m, 3H, Flu; 5H, PhSi), 4.32 (s, 1H, Flu), 1.62 (s, 6H, C(CH_3_)_2_), 1.15 (s, 1H, NH), 1.11 (s, 18H, Flu-t-Bu), 0.08 (s, 3H, SiCH_3_).

#### 2.2.2. Synthesis of (Diphenyl)silylene-Bridged Ligand L2

The ligand L2 was synthesized in a similar way to that for the synthesis of ligand L1 in good yield (76%).

^1^H NMR (CDCl_3_, 7.26): 8.61 (d, 1H, Naph), 7.78 (d, 1H, Naph), 7.67 (t, 1H, Naph; 3H, Flu), 7.45 (d, 1H, Naph; 2H, Flu), 7.37 (m, 1H, Flu; 3H, Naph), 7.31 (m, 6H, Ph), 7.20 (d, 3H, Ph), 7.11 (s, 1H, Ph), 3.97 (s, 1H, Flu), 1.59 (s, 3H, CCH_3_), 1.53 (s, 3H, CH_3_), 1.22 (s, 9H, Flu-tBu), 1.20 (s, 9H, Flu-tBu), 1.17 (s, 1H, NH).

#### 2.2.3. Synthesis of (Methylphenyl)silylene-Bridged Complex **1**

MeLi (1.6 M in ether, 15 mL, 24 mmol) was added dropwise at 0 °C into a solution of *ansa*-(MePh)silylene-(2,7-di-tBu)fluorenyl-naphthylamido ligand (3.49 g, 6 mmol) in 60 mL of diethylether. The resultant orange solution was stirred at r.t. for 4 h, then was added to a solution of TiCl_4_ (0.66 mL, 6 mmol) in 30 mL hexane at room temperature in the stirring condition for 2 h. The solvent was removed, and the residue was extracted with hexane (150 mL). The hexane solution was concentrated to 50 mL and cooled at −30 °C for several days to get complex **1** as red crystals (1.58 g, 40.1%).

^1^H NMR (CDCl_3_, 7.26): 8.77 (d, 1H, Naph), 7.98 (t, 2H, Flu), 7.82 (d, 1H, Naph), 7.75 (d, 1H, Naph), 7.71 (d, 2H, Flu), 7.66 (s, 1H, Ph), 7.59 (m, 2H, Flu), 7.49 (d, 1H, Naph), 7.39 (m, 3H, Naph), 7.34 (m, 3H, Ph), 7.24 (s, 1H, Ph), 2.31 (s, 3H, CCH_3_), 1.96 (s, 3H, CCH_3_), 1.39 (s, 9H, Flu-t-Bu), 1.15 (s, 9H, Flu-t-Bu), 0.32 (s, 3H, SiCH_3_), −0.40 (s, 3H, TiCH_3_), −0.28 (s, 3H, TiCH_3_). Anal. Calc. for C_43_H_51_NSiTi: C, 78.51; H, 7.81; N, 2.31. Found: C, 78.34; H, 7.88; N, 2.42.

#### 2.2.4. Synthesis of (Diphenyl)silylene-Bridged Complex **2**

Complex **2** was synthesized in a similar way to that for the synthesis of complex **1**, and dark red crystals were obtained in a 32% yield (1.38 g).

^1^H NMR (CDCl_3_, 7.26): 8.14 (d, 1H, Naph), 7.99 (t, 2H, Flu), 7.83 (d, 1H, Naph), 7.78 (d, 2H, Flu), 7.68 (m, 1H, Naph; 2H, 2Flu), 7.57 (d, 1H, Naph), 7.53 (s, 1H, Ph), 7.50~7.25 (m, 2H, Flu; 3H, Naph; 7H, PhSi), 6.86 (s, 1H, PhSi), 6.55 (s, 1H, PhSi), 1.29 (s, 3H, CCH_3_), 1.21 (s, 3H, CCH_3_), 1.34 (s, 9H, Flu-tBu), 1.09 (s, 9H, Flu-tBu), −0.23 (s, 3H, TiCH_3_), −0.41 (s, 3H, TiCH_3_). Anal. Calc. for C_48_H_53_NSiTi: C, 80.08; H, 7.42; N, 1.95. Found: C, 79.11; H, 7.48; N, 1.91.

### 2.3. Polymerization Procedure

The atmospheric pressure polymerization of propylene was performed in a 100 mL glass reactor equipped with a magnetic stirrer, and the high-pressure polymerization of propylene was carried out in a Parr Instrument Company autoclave (Moline, IL, USA). The reactor was vacuumed and charged with nitrogen three times before the polymerization procedure. Then, the reactor was charged with quantitative chlorobenzene (30 mL) and dMMAO solution under nitrogen, and polymerization was initiated by introducing the catalyst solution at the desired temperature and desired pressure. The polymerization was conducted for a specified time and terminated with acidic alcohol. The polymers obtained were washed by alcohol to remove dMMAO and ligand residue, and dried under vacuum at 80 °C for 6 h to a constant weight.

### 2.4. Analytical Procedure

A single crystal each from complexes **1** and **2** was mounted under nitrogen atmosphere at low temperature. The data collection was made on a Bruker APEX2 diffractometer (Karlsruhe, Germany) using graphite monochromated with Mo Ka radiation (λ = 0.71073 Å). The SMART program package was used to determine the unit cell parameters. The absorption correction was applied using the SADABS program [36]. All structures were solved by direct methods and refined on F^2^ by full-matrix least-squares techniques with anisotropic thermal parameters for non-hydrogen atoms. Hydrogen atoms were placed at calculated positions and were included in the structure calculation. Calculations were carried out using the SHELXS-97, SHELXL-2014, or Olex2 program [37,38,39,40,41,42]. Crystallographic data are summarized in Table 1, and CIF files are provided in the Supplementary Materials. However, disorder modeling of the butyl group for complex **1** was observed, and the ratio of the occupancies was fixed at 60:40; it was not refined by means of free variable for any reason.

Molecular weight and molecular weight distribution of the polymers were determined by a polymer laboratory PL GPC-220 (Santa Clara, CA, USA) with one guard column (PL# 1110–1120) and two 30 cm columns (PLgel 10 μm MIXED-B 7.5 × 300 mm). Polymer characterization was carried out at 150 °C using 1,2,4-trichlorobenzene as the eluent and calibrated by polystyrene standards. All ^13^C NMR spectra were recorded on a Bruker-600 spectrometer (Karlsruhe, Germany) at ambient temperature unless otherwise indicated. The chemical shifts of the ^13^C HMR spectra are referenced to the carbon resonance of 1,1,2,2-tetrachloroethane-d2 (δ: 74.47). DSC analyses were performed on a TA differential scanning calorimeter Q2000 (New Castle, DE, USA) and the DSC curves of the samples were recorded under nitrogen atmosphere at a heating rate of 10 °C/min from −30 to 220 °C.

## 3. Results and Discussion

### 3.1. Molecular Structure of Complexes

**Figure 1:**
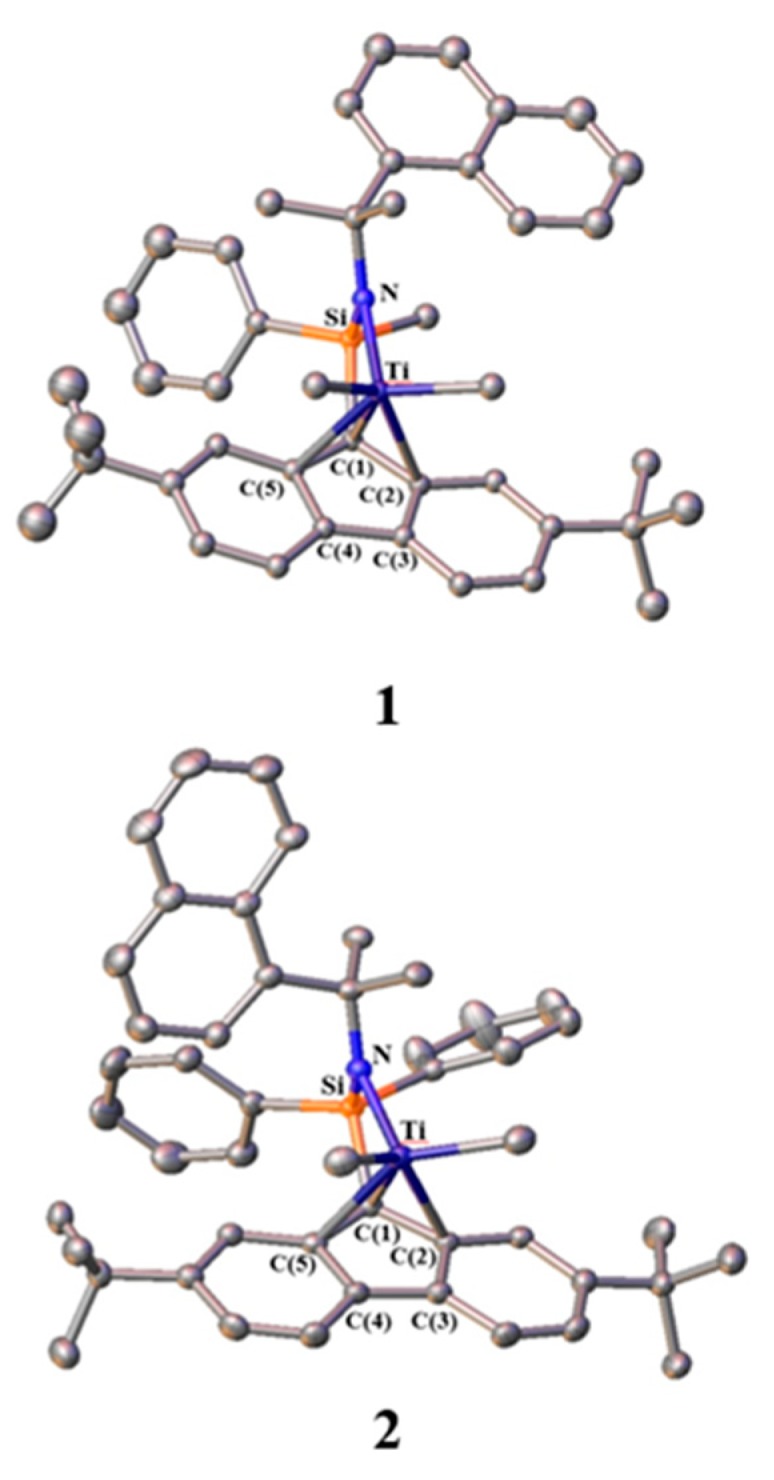
Molecular structures of complexes **1** and **2** (hydrogen atoms are omitted for clarity). Atoms are drawn at the 50% probability level. The disorder and solvent are not shown for complex **1** for the sake of clarity.

The (methylphenyl)silylene-bridged complex **1** and (diphenyl)silylene-bridged complex **2** were synthesized using a one-pot reaction of the corresponding ligand with a 2-fold excess of MeLi and TiCl_4_ in hexane. As compared to the ^1^H NMR spectrum of complex **F** reported previously, complexes **1** and **2** showed that the methyl groups bonded to Si and Ti atoms are non-equivalent, indicating that the phenyl substituent on the silylene bridge should affect the rotation of the naphthyl amido ligand to keep the *C*_1_-symmetric nature in solution. The molecular structures of **1** and **2**, as determined by single crystal X-ray analysis, are shown in Figure 1, and the selected bond lengths and angles of complexes are shown in Table 2.

In complexes **1** and **2**, the bond lengths between the titanium and fluorenyl carbons C(1), C(2), and C(5) were 2.267–2.449 and are similar to those lengths of previously reported [t-BuNSiMe_2_(C_5_Me_4_)]TiMe_2_ (**A**: Mt = Ti; R = Me, Scheme 1), in which the cyclopentadienyl ligand coordinates to the titanium metal with an η^5^-form [43]. Longer bond lengths (2.525–2.638) between the titanium and the fluorenyl carbons C(3) and C(4) were observed. The results indicate that the fluorenyl ligand is coordinating to the titanium metal with an η^3^- to η^1^-form irrespective of the structure of the substituent. In each molecular structure, the lack of a symmetric plane or axis also confirmed the *C*_1_-symmetric nature of **1** and **2** in the solid state.

### 3.2. Propylene Polymerization

Propylene polymerization was conducted using complexes **1** and **2** activated by dMMAO using a semi-batch method under different propylene pressures at 20 °C. The results are summarized in Table 3. The activity strongly depended on the silylene-bridged substituent and propylene pressure. In contrast to the results obtained with complex **F**, which contains a dimethyl silylene bridge, under an atmosphere pressure of propylene, complexes **1** and **2** were inactive for propylene polymerization, because of the steric hindrance effect of the phenyl group on the silylene bridge (entries 1 and 2, Table 3). The increase of the propylene pressure significantly improved the catalytic activities of complexes (up to 600 kg mol^−1^ h^−1^), affording high molecular weight polypropylene (*M*_n_ value up to 500 kg mol^−1^). Diphenyl silylene-bridged complex **2** exhibited much lower activity than that of (methylphenyl)silylene-bridged complex **1**, also indicating that the sterically bulkier phenyl substituent prevents the rotation of the naphthyl amido ligand, resulting in steric hindrance on the metal center.

**Figure 2:**
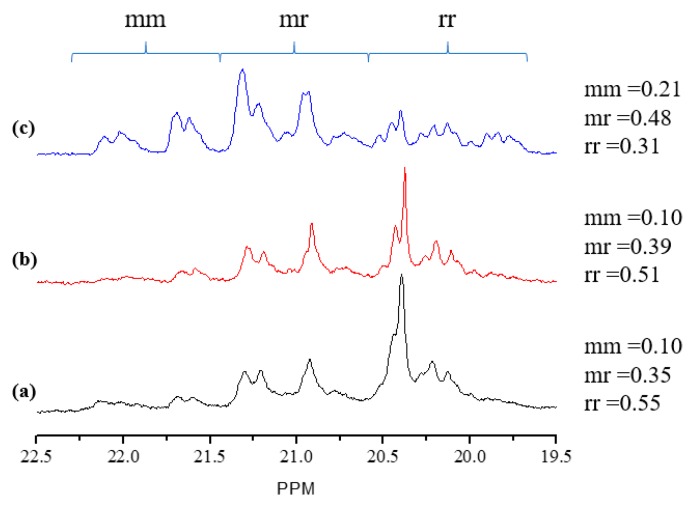
^13^C NMR spectra of the methyl region of polypropylene obtained with **1** ((**a**), entry 7), **2** ((**b**), entry 8), and **F** ((**c**), entry 9) in Table 3.

To investigate the structure-stereospecificity relationships of propylene polymerization using complexes **1** and **2**, the microstructures of polypropylene were measured by ^13^C NMR analysis. The ^13^C NMR spectra of the methyl region of polypropylene obtained with **F**, **1**, and **2** under a propylene pressure of 8.0 atm are shown in Figure 2, with the steric triad distributions in the main chain. As compared to the statistically atactic PP obtained with **F**, unexpected results were observed: **1** and **2** produced syndiotactic-rich polypropylene rather than isotactic PP, with rr values of 0.55 and 0.51, respectively, although complexes **1** and **2** display a *C*_1_-symmetric nature, as described by ^1^H NMR and X-ray crystal analyses. Consequently, all polymers obtained were amorphous, with the similar *T*_g_ value of −4 °C, regardless of the structure of the complex used.

### 3.3. Mechanical Properties of Polymers

**Figure 3:**
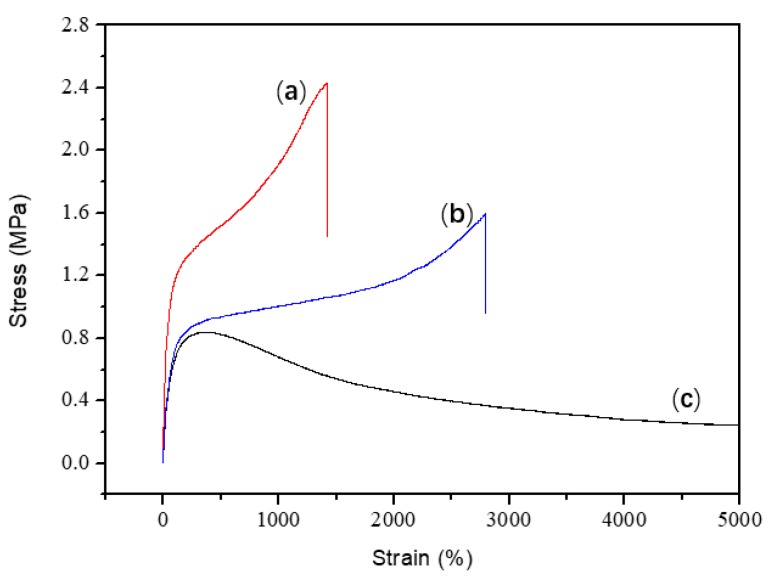
Stress–strain curves of selected polymers obtained with **1** ((**a**), entry 7), **2** ((**b**), entry 8), and **F** ((**c**), entry 9) in Table 3.

**Figure 4:**
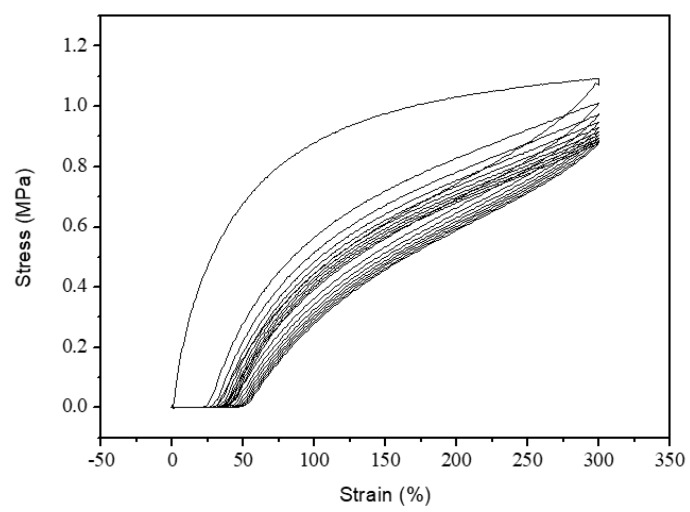
Repetitive stress–strain 10 cycles for polypropylene obtained with **1** (entry 1, Table 3).

To investigate the effect of the microstructure on the mechanical properties of the polypropylenes, tensile strength was measured for the polypropylene samples obtained with **F**, **1**, and **2** under a propylene pressure of 8.0 atm. The strain–stress curves are shown in Figure 3. The polymers obtained with **1** and **2** exhibited tensile strength values of 2.4 MPa and 1.6 MPa and elongation at break values of 1500–2800%, respectively. The elastic properties of the polymer obtained with **1**, which showed the highest tensile strength value, was then investigated. The sample was cyclically loaded and unloaded 10 times to 300% strain (Figure 4). The sample displayed better elastomeric properties after the first stress–strain cycle, with a certain amount of unrecovered strain due to a permanent structural change. For comparison, the polymer generated by complex **F** showed a very low tensile strength value (0.86 MPa) without elastic properties (elongation at break value of 5000%, instrument detection limit). The results indicate that the mechanical properties of the polypropylene samples are affected by their microstructures, since they showed almost the same molecular weight.

## 4. Conclusions

In conclusion, two novel *ansa*-silylene(fluorenyl)(amido) dimethyl titanium complexes **1** and **2** with a phenyl substituent on the silylene bridge were synthesized and characterized by ^1^H NMR, elemental analysis, and X-ray crystal analysis. The complexes displayed a *C*_1_-symmetric nature in both solution and solid state, as can be determined by the ^1^H NMR and X-ray analyses, respectively. Complex **1** containing the (methylphenyl)silylene bridge showed higher activity than (diphenyl)silylene-bridged complex **2** in propylene polymerization. The increase of propylene pressure was effective for enhancing polymerization activity (up to 600 kg mol^−1^ h^−1^) to produce high molecular weight polypropylene. The modification of the silylene bridge was not efficient for the isospecificity of a CGC catalyst, affording syndiotactic-rich polypropylene with an rr value of 0.50. The microstructure of polypropylene influenced the mechanical properties of the resulting polymers.

## Figures and Tables

**Table 1:** Crystallographic data and parameters for **F**, **1**, and **2.**

Complex	1	2
	CCDC 1539497	CCDC 1866644
moiety formula	C_46_H_58_NsiTi, 0.5(C_6_H_14_)	C_48_H_53_NSiTi
sum formula	C_46_H_58_NSiTi	C_48_H_53_NSiTi
formula weight	700.92	719.90
crystal system	Triclinic	Monoclinic
space group	*P* $\overset{-}{1}$	*P*1 2_1_/c1
a (Å)	10.3068(10)	12.3795(12)
b (Å)	13.0737(13)	9.9527(10)
c (Å)	15.1704(15)	32.554(3)
α (deg)	98.411(2)	90
β (deg)	94.191(2)	91.065(2)
γ (deg)	93.938(2)	90
V (Å^3^)	2010.3(3)	4010.3(7)
Z	2	4
F (000)	754	1536
density(calcd.) (g·cm^−3^)	1158	1192
absorption coefficient μ (mm^−1^)	0.274	0.277
theta range for data collection	1.922 to 25.497°	1.251 to 28.362°
reflections collected	14,202	33,691
independent reflections	7483	9964
final R indices [I > 2δ(I)]	[R(int) = 0.0258]	[R(int) = 0.0917]
	R1 = 0.0389	R1 = 0.0556
	wR2 = 0.0965	wR2 = 0.1060

**Table 2:** Selected bond lengths (Å) and bond angles [deg] for related complexes.

Parameter	F [35]	1	2
Ti–C(1)	2.267(3)	2.238(17)	2.245(3)
Ti–C(2)	2.413(3)	2.449(18)	2.441(3)
Ti–C(3)	2.576(3)	2.638(18)	2.570(3)
Ti–C(4)	2.569(3)	2.595(17)	2.525(2)
Ti–C(5)	2.394(3)	2.347(17)	2.370(2)
Ti(1)–N(1)	1.920(2)	1.934(15)	1.954(2)
Ti(1)–Si(1)	2.848(11)	2.846(6)	2.833(9)
N(1)–Ti(1)–C(1)	77.74(11)	77.99(6)	77.41(9)
Ti(1)–N(1)–Si(1)	101.76(12)	100.89(7)	100.10(10)
N(1)–Si(1)–C(1)	93.88(13)	93.32(7)	94.27(11)

**Table 3:** Propylene polymerization with Ti complexes **F**, **1**, and **2** ^a^.

Entry	Catalyst	Pressure (atm)	Time (min)	Yield (g)	Activity (kg mol^−^^1^ h^−^^1^)	*M*_n_^b^ (×10^4^)	*M*_w_/*M*_n_^b^	*T*_m_^c^ (°C)	*T*_g_^c^ (°C)
1	**1**	1	12	0	0	-	-	-	-
2	**2**	1	12	0	0	-	-	-	-
3 ^d^	**F**	1	12	0.36	91	4.38	1.27	- ^e^	−4.3
4	**1**	4	20	0.54	162	25.7	2.18	- ^e^	−4.0
5	**2**	4	20	0.18	54	13.2	2.37	- ^e^	−3.3
6	**F**	4	20	1.20	360	34.8	2.42	- ^e^	−4.6
7	**1**	8	20	1.99	597	54.8	2.14	- ^e^	−3.7
8	**2**	8	20	0.62	186	49.3	2.05	- ^e^	−4.0
9	**F**	8	20	2.34	702	60.1	1.89	- ^e^	−3.4

^a^ Polymerization conditions: Chlorobenzene = 30 mL, Ti = 10 μmol, dried modified methyaluminoxane (dMMAO) = 4.0 mmol, polymerization temperature = 20 °C; ^b^ Molecular weight and molecular weight distribution were determined by GPC using polystyrene standards; ^c^ Melting point and glass transition temperature were determined by DSC. ^d^ Data taken from [35]; ^e^ Not detected.

## Supplementary Materials

The following supplementary materials are available online at http://www.mdpi.com/2073-4360/10/10/1075/s1.

## References

[1] Brintzinger H.H., Fischer D., Mülhaupt R., Rieger B., Waymouth R.M. (1995). Stereospecific olefin polymerization with chiral metallocene catalysts. Angew. Chem. Int. Ed. Engl..

[2] Bochmann M. (1996). Cationic group 4 metallocene complexes and their role in polymerization catalysis: The chemistry of well defined Ziegler catalysts. J. Chem. Soc. Dalton Trans..

[3] Resconi L., Cavallo L., Fait A., Piemontesi F. (2000). Selectivity in prpene polymerization with metallocene catalysts. Chem. Rev..

[4] Alt H.G., Koppl A. (2000). Effect of the nature of metallocene complexes of group Ⅳ metals on their performance in catalytic ethylene and propylene polymerization. Chem. Rev..

[5] Coates G.W. (2000). Precise control of polyolefin stereochemistry using single-site metal catalysts. Chem. Rev..

[6] Ittel S.D., Johnson L.K., Brookhart M. (2000). Late-metal catalysts for ethylene homo- and copolymerization. Chem. Rev..

[7] Coates G.W., Hustad P.D., Reinartz S. (2002). Catalysts for the living insertion polymerization of alkenes: Access to new polyolefin architectures using Ziegler-Natta chemistry. Angew. Chem. Int. Ed..

[8] Gibson V.C., Spitzmesser S.K. (2003). Advances in non-metallocene olefin polymerization catalysis. Chem. Rev..

[9] Makio H., Terao H., Iwashita A., Fujita T. (2011). FI catalysts for olefin polymerization—A comprehensive treatment. Chem. Rev..

[10] Mu H., Pan L., Song D., Li Y. (2015). Neutral nickel catalysts for olefin homo- and copolymerization: Relationships between catalyst structures and catalytic properties. Chem. Rev..

[11] Pamela J., Shapiro E.B., William P.S., John E.B. (1990). [{(η^5^-C_5_Me_4_)Me_2_Si(η^1^-NCMe_3_)}(PMe_3_)ScH]_2_: A unique example of a single-component α-olefin polymerization catalyst. Organometallics.

[12] Okuda J., Schattenmann F.J., Wocadlo S., Massa S.W. (1995). Synthesis and characterization of zirconium complexes containing a linked amido-fluorenyl ligand. Organomtallics.

[13] Waymouth R.M., Andrem L.K. (1998). Group 4 *ansa*-cyclopentadienyl-amido catalysts for olefin polymerization. Chem. Rev..

[14] Xu G. (1998). Copolymerization of ethylene with styrene catalyzed by the [η^1^:η^5^-tert-butyl (dimethylfluorenylsilyl)amido]methyltitanium “cation”. Macromolecules.

[15] Irwin L.J., Reibenspies J.H., Miller S.A. (2004). A sterically expanded “constrained geometry catalyst” for highly active olefin polymerization and copolymerization: An unyielding comonomer effect. J. Am. Chem. Soc..

[16] Schwerdtfeger E.D., Miller S.A. (2007). Intrinsic branching effects in syndiotactic copolymers of propylene and higher α-olefins. Macromolecules.

[17] Schwerdtfeger E.D., Irwin L.J., Miller S.A. (2008). Highly branched polyethylene from ethylene alone via a single zirconium-based catalyst. Macromolecules.

[18] Schwerdtfeger E.D., Price C.J., Chai J., Miller S.A. (2010). Tandem catalyst system for linear low-density polyethylene with short and long branching. Macromolecules.

[19] Chai J., Khalil A.A., Miller S.A. (2013). Sterically expanded CGC catalysts: Substituent effects on ethylene and α-olefin polymerization. Dalton Trans..

[20] Jung H.Y., Hong S.D., Jung M.W., Lee H., Park Y.W. (2005). Norbornene copolymerization with α-olefins using methylene-bridge *ansa*-zirconocene. Polyhedron.

[21] Kirillov E., Razaci A., Carpentier J.F. (2006). Syndiotactic-enriched propylene-styrene copolymers using fluorenyl-based half-titanocene catalysts. J. Mol. Catal. A.

[22] Na S.J., Wu C.J., Yoo J., Kim B.E., Lee B.Y. (2008). Copolymerization of 5,6-dihydrodicyclopentadiene and ethylene. Macromolecules.

[23] Yu S.T., Na S.J., Lim T.S., Lee B.Y. (2010). Preparation of a bulky cycloolefin/ethylene copolymer and its tensile properties. Macromolecules.

[24] Nakayama Y., Sogo Y., Cai Z., Shiono T. (2013). Copolymerization of ethylene with 1,1-disubstituted olefins catalyzed by *ansa*-(fluorenyl)(cyclododecylamido)dimethyltitanium complexex. J. Polym. Sci. Part A Polym. Chem..

[25] Razavi A., Thewalt U. (2001). Preparation and crystal structures of the complexes (η^5^-C_5_H_3_TMS-CMe_2_-η^5^-C_13_H_8_)MCl_2_ (M=Hf, Zr or Ti): Mechanistic aspects of the catalytic formation of a isotactic-syndiotactic stereoblock-type polypropylene. J. Organomet. Chem..

[26] Busico V., Cipullo R., Cutillo F., Talarico G., Razavi A. (2003). Syndiotactic poly(propylene) from [Me_2_Si(3,6-di-tert-butyl-9-fluorenyl)(N-tert-butyl)]TiCl_2_-based catalysts: Chain-end or enantiotopic-sites stereocontrol?. Macromol. Chem. Phys..

[27] Irwin L.J., Miller S.A. (2005). Unprecedented syndioselectivity and syndiotactic polyolefin melting temperature: Polypropylene and poly(4-methyl-1-pentene) from a highly active, sterically expanded η^1^-fluorenyl-η^1^-amido zirconium complex. J. Am. Chem. Soc..

[28] Nishii K., Ikeda T., Akita M., Shiono T. (2005). Polymerization of propylene with [*t*-BuNSiMe_2_Ind]TiMe_2_-MAO catalyst systems. J. Mol. Catal..

[29] Tanaka R., Chie Y., Cai Z., Nakayama Y., Shinon T. (2016). Structure-stereospecificity relationships of propylene polymerization using substituted *ansa*-silylene(fluorenyl)(amido)titanium complexes. J. Organomet. Chem..

[30] Shiono T. (2011). Living polymerization of olefins with ansa-dimethyl-silylene(fluorenyl)(amido) dimethyltitanium-based catalysts. Polym. J..

[31] Cai Z., Su H., Shiono T. (2013). Precise synthesis of olefin block copolymer using a syndiospecific living polymerization system. Chin. J. Polym. Sci..

[32] Cai Z., Ikeda T., Akita M., Shiono T. (2005). Substituent effects of tert-butyl groups on fluorenyl ligand in syndiospecific living polymerization of propylene with *ansa*-fluorenylamidodimethyl titanium complex. Macromolecules.

[33] Kleinschmidt R., Griebenow Y., Fink G. (2000). Stereospecific propylene polymerization using half-sandwich metallocene/MAO systems: A mechanistic insight. J. Mol. Catal. A Chem..

[34] Cai Z., Su H., Nakayama Y., Shiono T., Akita M. (2014). Synthesis of *C*_1_ symmetrical *ansa*-cyclopentadienylamidotitanium complexes and their application for living polymerization of propylene. J. Organomet. Chem..

[35] Wang H., Wang X., Sun Y., Cheng H., Shiono T., Cai Z. (2017). Living polymerization of propylene with *ansa*-dimethylsilylene(fluorenyl)(cumylamido) titanium complexes. Polymers.

[36] Sheldrick G.M. (1996). SADABS: An Empirical Absorption Correction Program for Area Detector Data.

[37] Sheldrick G.M. (2008). Crystal structure refinement with SHEXL. Acta Crystallogr. Sect. C.

[38] Sheldrick G.M. (2015). A short history of SHELX. Acta Crystallogr. Sect. A.

[39] Dolomanow O.V., Bourhis L.J., Gildea R.J., Howard J.A.K., Puschmann H. (2009). OLEX2: A complete structure solution, refinement and analysis program. J. Appl. Crystallogr..

[40] (2002). SAINT+.

[41] (2002). SAINT+.

[42] (2002). SHELXTL NT/2000.

[43] Ioku A., Hasan T., Shiono T., Ikeda T. (2002). Effects of cocatalysts on propene polymerization with [*t*-BuNSiMe_2_(C_5_Me_4_)]TiMe_2_. Macromol. Chem. Phys..

